# Integrated actions of assistance to the diabetic patient

**DOI:** 10.1186/1758-5996-7-S1-A178

**Published:** 2015-11-11

**Authors:** Danielle Rachel dos Santos Carvalho, Márcia Alves de Souza, Francisco Geraldo Mello da Rocha Carvalho Neto, Simone Bruggemann Mota, Adolfo José da Mota, Tetsuo Yamane, Deborah Laredo Jezini

**Affiliations:** 1 Universidade Federal do Amazonas – UFAM, Manaus, Brazil

## Background

WHO alerts to the epidemic behavior of diabetes (DM): more than 190 million people worldwide suffer from diabetes and this number will double by 2030 (Brasilsus, 2014). In Brazil, IDF estimated in 2014 that the number of diabetics was 11.6 million. Aging, urbanization, sedentary lifestyle, high fat diet and obesity are seen as responsible for this situation. In addition to the cost of treatment, complications of the syndrome, overtax and overwhelm the health care system. Thus, only public policies are insufficient, being essential the efforts of researchers and health teams to carry out projects for the guidance of the population, who often have no basic knowledge, compromising the effectiveness of treatment.

## Objectives

Carry out actions, especially for the elderly, involving students in the selection and placement of information about etiology, symptoms, diagnosis, treatment, side effects and drug interactions of DM.

## Materials and methods

This project is part of an extension program with continued actions, involving 20 volunteers students, per event, and Uninorte and UFAM teachers. The activities have multivariate approaches, are cyclical and redundant, ensuring maximum participants. In the first half of 2015, the subjects were: pharmaceutical and nutritional guidance, physical activity and risks of alcohol consumption and smoking. Diabetic people interested in the Monitoring Group, scheduled for Sep/2015, were registered. These people will receive glucometer and a data collection form for monitoring capillary blood glucose and receive guidelines. Also will be offered, the molecular test for the registered ones with a possible diagnosis of MODY 2 or 3 (Ethical approval n° 923 744).

## Results

The activities took place in the Dr. Thomas Foundation and Senior Park/Vieiralves, Manaus, AM. 70 people were served. Of these, 4 diabetic female, have registered. The accession of the elderly, especially women is a goal, as they generally are more receptive to information, make appropriate medical monitoring, influencing your partner and being an example to children and grandchildren.

## Conclusion

The project confirms the importance of university extension for the formation of a professional who recognizes in their proactive actions a way to benefit the community. The exchange of experiences promote changes in habits and learning, whose expected benefit is to prevent and/or minimize the complications of diabetes by improving the quality of life.

